# The efficiency of detecting seabird behaviour from movement patterns: the effect of sampling frequency on inferring movement metrics in Procellariiformes

**DOI:** 10.1186/s40462-024-00499-1

**Published:** 2024-09-02

**Authors:** Stefan Schoombie, Rory P. Wilson, Yan Ropert-Coudert, Ben J. Dilley, Peter G. Ryan

**Affiliations:** 1 DST-NRF Centre of Excellence, FitzPatrick Institute of African Ornithology, University of Cape Town, Rondebosch, 7701 South Africa; 2 Department of Statistical Sciences, Centre for Statistics in Ecology, Environment and Conservation (SEEC), University of Cape Town, Cape Town, 7701 South Africa; 3 Department of Biosciences, Swansea University, Swansea, SA1 8PP UK; 4 Centre d’Etudes Biologiques de Chizé, Station d’Écologie de Chizé-La Rochelle Université, CNRS UMR7372, Villiers-en-Bois, France

**Keywords:** Albatross, Tracking, GPS, GNNS, Error rates, State-space model, Behaviour

## Abstract

**Background:**

Recent technological advances have resulted in low-cost GPS loggers that are small enough to be used on a range of seabirds, producing accurate location estimates (± 5 m) at sampling intervals as low as 1 s. However, tradeoffs between battery life and sampling frequency result in studies using GPS loggers on flying seabirds yielding locational data at a wide range of sampling intervals. Metrics derived from these data are known to be scale-sensitive, but quantification of these errors is rarely available. Very frequent sampling, coupled with limited movement, can result in measurement error, overestimating movement, but a much more pervasive problem results from sampling at long intervals, which grossly underestimates path lengths.

**Methods:**

We use fine-scale (1 Hz) GPS data from a range of albatrosses and petrels to study the effect of sampling interval on metrics derived from the data. The GPS paths were sub-sampled at increasing intervals to show the effect on path length (i.e. ground speed), turning angles, total distance travelled, as well as inferred behavioural states.

**Results:**

We show that distances (and per implication ground speeds) are overestimated (4% on average, but up to 20%) at the shortest sampling intervals (1–5 s) and underestimated at longer intervals. The latter bias is greater for more sinuous flights (underestimated by on average 40% when sampling > 1-min intervals) as opposed to straight flight (11%). Although sample sizes were modest, the effect of the bias seemingly varied with species, where species with more sinuous flight modes had larger bias. Sampling intervals also played a large role when inferring behavioural states from path length and turning angles.

**Conclusions:**

Location estimates from low-cost GPS loggers are appropriate to study the large-scale movements of seabirds when using coarse sampling intervals, but actual flight distances are underestimated. When inferring behavioural states from path lengths and turning angles, moderate sampling intervals (10–30 min) may provide more stable models, but the accuracy of the inferred behavioural states will depend on the time period associated with specific behaviours. Sampling rates have to be considered when comparing behaviours derived using varying sampling intervals and the use of bias-informed analyses are encouraged.

**Supplementary Information:**

The online version contains supplementary material available at 10.1186/s40462-024-00499-1.

## Background

Understanding where, when, and how free-ranging animals spend their time is important for effective conservation of their populations [1, 2] as well as the management of conflicts between humans and wildlife [3, 4]. Seabirds forage in an environment where resources are sparsely distributed across a vast area [5] but, while breeding, they are central place foragers, constrained to return to their nesting grounds to incubate eggs or feed chicks. This makes them an interesting group of birds to study as they all start at the same point, but do not necessarily target the same foraging areas (e.g. [6, 7]) The albatrosses and petrels (Procellariiformes) are among the most extreme central place foragers as they can travel at great speeds and remain away from their nests for up to 2–4 weeks while incubating eggs or provisioning chicks [8–13]. However, their far-ranging nature puts them at risk of overlapping with fishing operations, and indeed many Procellariiformes are threatened by mortality on fishing gear [14]. Procellariiformes are top-predators in the Southern Ocean where they are potential indicators of ecosystem health [2] and studying their movement and behaviour is crucial for conservation of biodiversity within the Southern Ocean [15].

Studying the behaviour of far-ranging marine animals is difficult to do as direct observation is seldom possible. The introduction of microprocessors in bio-logging technology in the 1980s started a revolution of new ways to study animals in their natural environment [16, 17]. This includes satellite tracking, which is a form of telemetry used to determine the location of an animal anywhere on the planet, as long as the device is not obstructed (i.e. under water or dense tree cover; [18]). This is especially useful for wide-ranging or air-breathing marine organisms that cannot easily be tracked with other technologies, such as VHF radio tags [19], which may require extensive networks of base stations or receivers. From the mid-1990s, seabirds were most often tracked with platform terminal transmitters (PTTs) using Argos satellites to triangulate bird locations every 1–3 h to a precision of a few kilometres [20]. In the last two decades the use of global positioning system (GPS) devices are preferred to PTTs as they are more accurate (± 5 m; [21–24]), can record locations more frequently [15, 25] and do not need to be retrieved when used in conjunction with Argos or GSM systems (Global System for Mobile Communications) to transmit data [26, 27]. In the past, studies using GPS/PTT devices to track animals suffered from small sample sizes as a result of high device costs, device failures and loss [25]. However, recent technological advances and increased demand in the electronics consumer sector [17] have driven reductions in device costs and increased reliability, resulting in larger datasets that detail the movement of top predators in the Southern Ocean [2, 6].

Many studies of animal behaviour use a combination of path length and turning angle to segregate GPS location estimates of a foraging trip into discrete states using state space models [28] or path segmentation [29]. Among state space models, Hidden-Markov Models (HMM) have shown great promise when inferring behavioural states [30] which are often split into resting, commuting, and foraging. This is particularly useful for far-ranging or hard to observe animals, such as seabirds, but validation of these models is mostly done with sampling rates of 1–10 min [30–32] and although we know that path geometries used in these models are sensitive to sampling rate [33], few empirical studies have addressed the effects of sampling rate, especially at finer scales. We know that seabird behaviour at sea is scale-dependent [21, 34, 35] and that sampling rates may affect metrics derived from GPS locations, such as ground speed [36]. However, few studies have examined the effect of sampling frequency on detected movement rates [22, 37] and to our knowledge, none of these studies have included flying seabirds [25].

The study of seabird movement using GPS locations has increased dramatically in recent years and studies collating large datasets from various sources are becoming more popular [6, 38–41]. However, a wide range of sampling intervals are used in these tracking studies. For example, albatross foraging behaviour has been studied using fine-scale tracking data with sampling rates < 10 s [42, 43], intermediate rates (1–15 min; [44]) as well as coarse sampling rates > 15-min [6, 45–47]. Studies with varying sampling intervals in their data usually sub-sample the data to the coarsest rate [6] or analyse data with similar rates separately [45], but the explicit effect of sampling rates on the inferred metrics is rarely acknowledged, let alone assessed. Here, we refer to these effects as biases, rather than errors, as the latter term is typically used to describe measurement error on the location estimates when working with tracking data. Although bias informed analytical tools are now available to compare datasets with varying sampling rates [48], current research still uses conventional methods (e.g. [49]) and defining biases associated with such methods is essential for comparison with historical data.

In ideal circumstance, when studying animal behaviour from tracking data, the objective of the research should dictate the sampling rate and not the other way around [50]. This can be problematic as high sampling rates usually require large power sources and are impractical for behaviours that stretch over extended periods (e.g. distance travelled during a foraging trip). However, location estimates at coarse sampling rates might not be applicable to the temporal scale of particular behaviours, and behavioural states [51], spatial distribution [37], and social behaviours [52] identified from location-based data might vary with sampling intervals. Although GPS loggers can acquire data at infrasecond rates [53, 54], low-cost GPS loggers typically contain a SIRFstarIII chipset with a maximum update rate of 1 Hz [23]. However, most seabird tracking studies use even less frequent sampling intervals, mainly to conserve battery life but also to prevent spurious movement estimates arising from positional errors [22, 54]. Battery life is particularly important for far-ranging seabirds that undertake foraging trips lasting many days as the logger mass (and consequently battery size) is constrained by the size of the bird. Empirical studies suggest that a seabird’s behaviour is adversely affected if a logger weighs > 2–3% of the bird’s mass [55]. Most studies of seabird movements using GPS loggers prioritize battery life rather than frequent sampling rates to ensure data for at least one complete foraging trip (e.g. [6]). Advances in microelectronic technology have reduced the size of GPS loggers allowing smaller seabird species to be studied using this technology [56], but again the effective lifespan of these loggers is limited by battery mass. It is often suggested that when comparing behaviours derived from several sampling scales, the coarser scale should be used for all data [57]. However, reducing location-based sampling intervals can limit the inferences that can be made from the data and advances in analytical tools may allow varying sampling rates under certain conditions [48]. Coarse sampling rates may be appropriate to study large-scale distributions of animals, but such rates might be less useful when inferring habitat selection or animal behavior from the same data [57, 58] and clear guidelines are necessary to inform researchers when designing studies or interpreting existing data [52].

When using GPS data to infer movement patterns, two main errors related to sampling rates are of concern: measurement error and interpolation error [59, 60]. Measurement error occurs when too frequent sampling overestimates distances covered whereas interpolation error occurs at coarse sampling rates with large gaps between points, underestimating actual path length [59, 60]. Studies where fine-scale (< 10 s sampling rate) GPS data are used to estimate biases associated with these errors, are more frequently found in industrial applications where size constraints do not apply [60]. In this study we present a dataset of fine-scale (1 Hz) GPS paths from a range of procellariiform species. By sub-sampling the fine-scale data, we quantify the effects of biases associated with low-cost GPS logger at varying sampling rates. Lastly, we show an example of how classification outcomes can change when data with varying sampling rates are used and describe best practice to address ecological questions using GPS data.

## Methods

### Field sampling

The development of satellite tracking technology is ongoing and newer devices are now able to process data from several satellite systems (in addition to the conventional GPS system) and are referred to as global navigation satellite system (GNSS) devices [26]. The loggers we used only logged location estimates from GPS satellites and for simplicity, when referring to satellite tracking, we use the term GPS. Data from several albatross and petrel species breeding on Gough (40° 20′ S, 9° 50′ W), Nightingale (37° 25′ S, 12° 28′ W; Tristan da Cunha archipelago), and Marion Islands (46° 50′ S, 37° 50′ E) were collected between 2014 and 2020 using CatTraQ (Catnip Technologies, Ltd 2013) or i-gotU (Mobile Action Technology, Inc. 2013) GPS loggers recording positions every second (except for one bird with a 2-s sampling interval; Table 1). These were near identical GPS units, with a SiRFstarIII chipset and a ceramic patch antenna with a maximum update rate of 1 Hz. The GPS loggers were programmed to start a few days after deployment to maximize the likelihood of obtaining at-sea points, or loggers were deployed on individuals that had just been relieved by their partner at their nest. The loggers were 42 × 26 × 10 mm with a 380 mAh battery, weighing 15.7 g and were attached to the backs of the birds with waterproof Tesa tape (Beiersdorf). For the smaller petrels, a 200 mAh battery was used, reducing the mass to 12.7 g. The combined device mass (including attachment tape and waterproofing) was well below 3% of body mass for albatrosses [61] and did not exceed 5% of mass for smaller petrels. It has to be noted that a 3–5% body mass threshold may differ between species [62]. Albatrosses are large birds and although it could not be explicitly tested, there was no indication that the devices had any adverse effects on the birds. The added mass of devices may have an effect on the performance of smaller petrels and the results from these individuals have to be considered with this in mind.

**Table 1:** Fine-scale GPS tracking data acquired from albatross and petrel species from three Southern Ocean breeding islands, listed in order of descending body mass

Species	Breeding island	Year	Number of individuals	Sampling rate (s)	Logging duration (mean hours ± SD)
Wandering albatross *Diomedea exulans*	Marion	2018	4	1	8 ± 6
		2020	4	1	25 ± 15
Tristan albatross *Diomedea dabbenena*	Gough	2014	2	1 & 2	15 ± 0
Grey-headed albatross *Thalassarche chrysostoma*	Marion	2019	1	1	8
Sooty albatross *Phoebetria fusca*	Marion	2019	2	1	25 ± 1
Atlantic yellow-nosed albatross *Thalassarche chlororhynchos*	Gough	2014	2	1	13 ± 6
Grey petrel	Nightingale	2014	2	1	15 ± 0
*Procellaria cinerea*	Gough	2016	2	1	12 ± 2
Great shearwater *Ardenna gravis*	Gough	2016	1	1	15
	Nightingale	2016	5	1	17 ± 1
Atlantic petrel *Pterodroma incerta*	Gough	2014	1	1	13
Soft-plumaged petrel *Pterodroma mollis*	Gough	2016	2	1	15 ± 0

### Reference distance

When sub-sampling our 1 Hz GPS data we needed to compare the resulting paths to some sort of ground-truth reference. Because we had fine-scale GPS data, we could use the instantaneous speed measurement of the GPS (also known as point speed) as a reference distance for each point. Point speed measurements recorded by GPS units are very accurate as they are inferred directly from the satellite signal using the Doppler effect [59, 63]. These point speeds are independent of measurement error and can be used as a reference distance when interpolation error is negligible (as is the case at a 1-s sampling interval; [60, 64]). It is important to note that we present the point speed measures as distance and thus the units are in metres, i.e. when the point speed is 10 m/s the reference distance would be 10 m at that point.

To test if point speed was indeed a valid reference distance for the loggers used in this study, 10 GPS loggers were placed in a stationary position with an unobstructed view of the sky and away from any structures on Marion Island. The loggers were placed outside on 19 July (3 loggers, ID S1–S3) and 21 July 2019 (7 loggers, ID S4–S10) and left to record until they ran out of battery power (∼ 12 h later). The mean ± SD total distance as well as the point speed recorded by the stationary loggers was calculated. A Welch two-sample *t*-test was used to test for significant differences between speed and location-derived distances reported by the stationary GPS loggers. Speed and distance were both log-transformed to conform to a normal distribution.

### Data analysis

#### Path preparation

The GPS data were cleaned prior to analysis by removing points with duplicate date and time values as well as on-land points (22412 points ∼ 2% of total). The cleaned data were converted to trajectories (referred to as paths) using the R package *adehabitatLT* [65]. The sinuosity of foraging trips was expected to increase with trip duration and thus we split the paths into individual flights (hereafter ‘flights’) to account for variation in foraging trips. Non-flying periods were identified where the average speed over 10 s was < 10 km·h^− 1^ for at least 60 s. These non-flying periods were removed and the subsequent breaks in data were used to define individual flights.

**Fig. 1:**
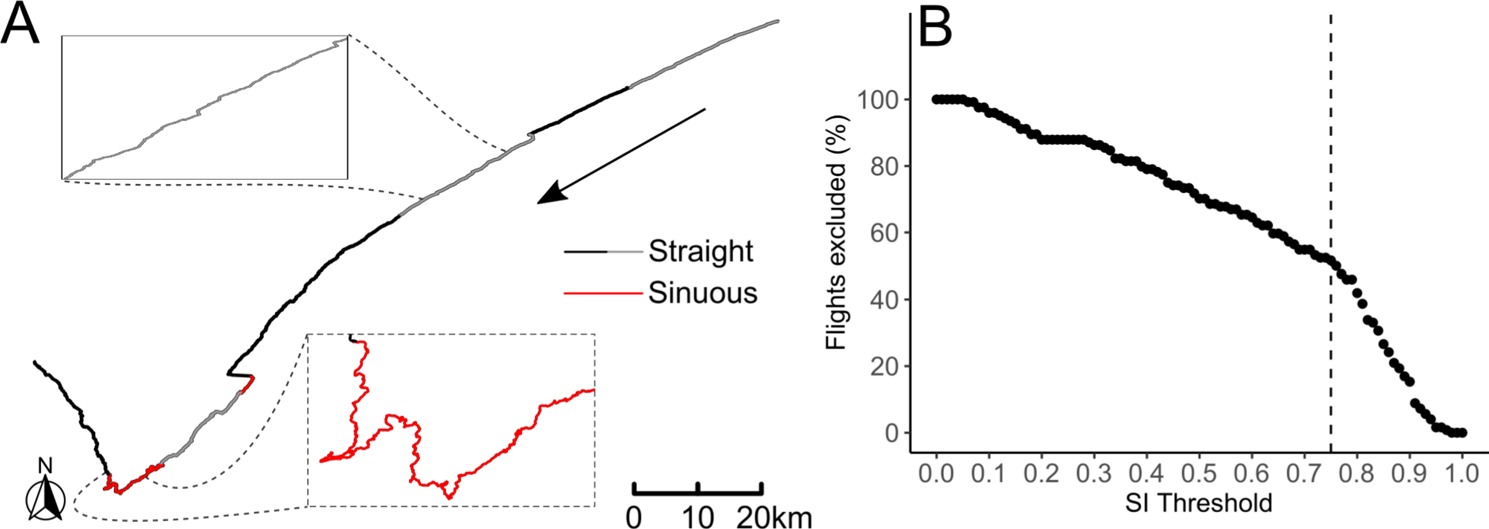
(**a**) Example of classifying a 1-s path from an Atlantic petrel as straight or sinuous flight as chosen by a straightness index (SI) threshold, identified in (**b**). The tracked path lasted ∼ 11 h. The arrow shows the direction of flight and alternating colours indicate individual flights

Our preliminary analysis showed that there was a clear distinction between different flight types, but seeing that our sampling duration was limited, some individuals had a disproportionate amount of a single flight type. Thus, we opted to group the flights into two groups based on their sinuosity. A straightness index (SI) was calculated for each flight by dividing the great-circle distance between the first and last points of the flight by the total distance travelled during the flight (i.e. values close to 1 represent a straight flight; Fig. 1a). A threshold value was used to classify flights as either straight or sinuous based on their SI, where straight flights had a SI larger than the threshold. To estimate an appropriate threshold value, a threshold (ranging from 0 to 1) was applied to all flights in a stepwise manner and the percentage of flights with a SI lower than the threshold was reported (Fig. 1b). The threshold value (0.75 in our case) was chosen where the resulting curve’s gradient changed significantly. A linear mixed-effects model with sinuosity as predictor and species as response was used to test for differences in means of sinuosity between species, where individual birds were added as a random effect. Although sinuous flights are often associated with foraging flights, we did not differentiate between commuting and foraging here as the sampling rates will affect the outcome of behavioural state estimates (see Results).

#### Sub-sampling

**Fig. 2:**
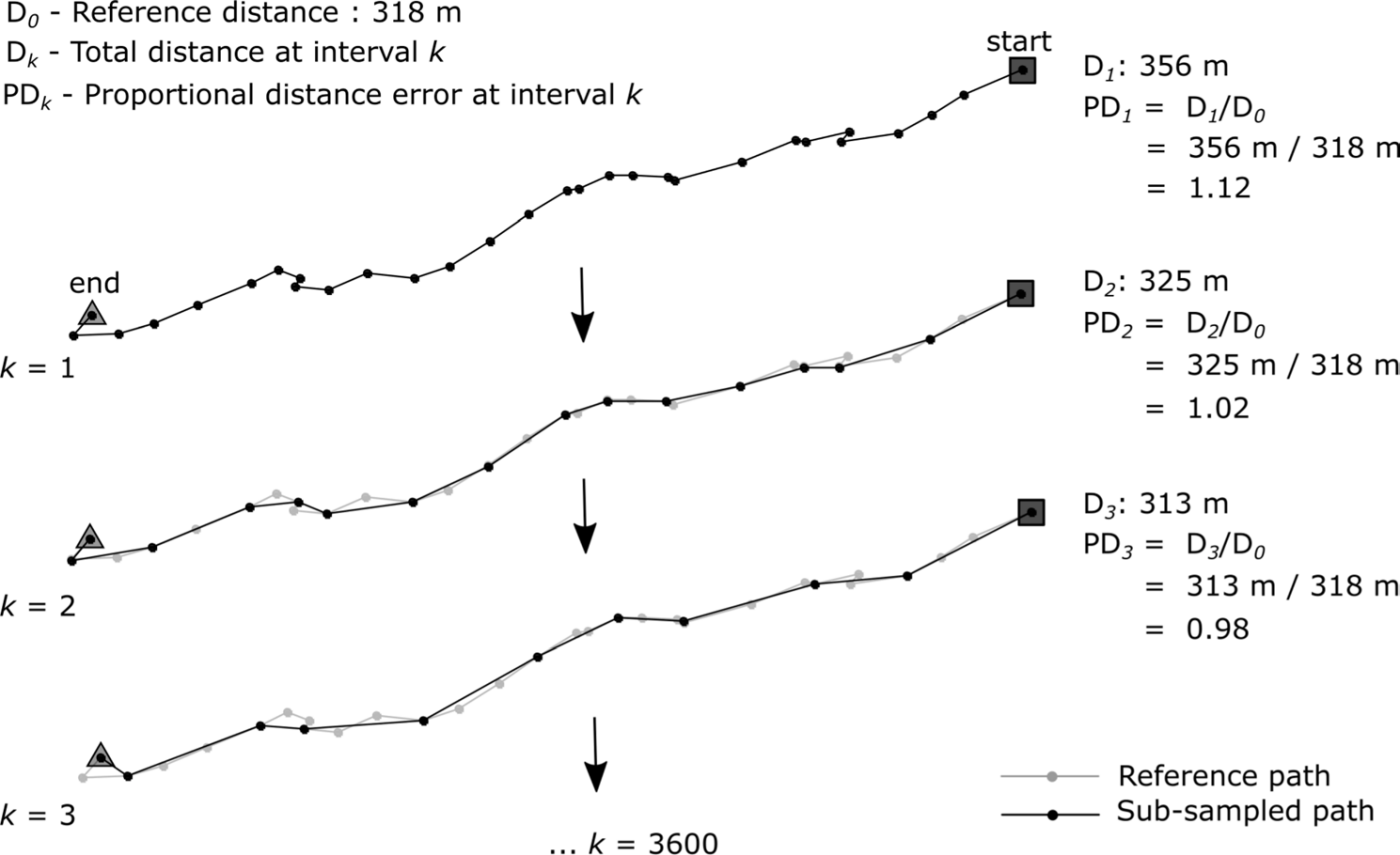
Steps followed when sub-sampling a flight to determine the proportional distance at varying sampling intervals (PD_*k*_). Shown here is a hypothetical 30 s flight with an actual path length (D_0_) of 318 m (as determined by point speed measurements)

To determine the effect of sampling rate on seabird GPS trajectories, the sampling interval of individual flights was increased at 1-s time intervals (*k*; sub-sampled flight denoted as *F*_*k*_) and compared to the reference flight (*F*_*0*_), as explained in [60]. One Tristan albatross had a sampling interval of 2-s (Table 1), for this individual the sampling interval was increased at 2-s intervals. Sub-sampling was done using the *redisltraj* function from the *adehabitatLT* package [65], with *k* up to 3600 s (1 h). Thus, only flights that were > 1 h were used for the analysis. *F*_*0*_ was equal to *F*_*1*_ except for distance estimates between points, where point speed was used as a proxy for distance in *F*_*0*_, while great circle distances between location estimates were used in *F*_*k*_. Point speeds proved to be more accurate (see Results) when determining path lengths at short intervals where path lengths from distance between consecutive points were subject to measurement error. Because our reference path *F*_*0*_ was at 1-s sampling interval, the point speed measurement (expressed in m/s) is equal to the path length for the reference path. Speed between sub-sampled points was calculated as the distance divided by the sampling interval *k.* The raw paths were set to record location at 1-s intervals, but for unknown reasons, where unable to maintain the 1-s regime and intermittently (∼ 10%; see Results) recorded location at 2-s sampling intervals. These points were linearly interpolated to obtain a uniform 1-s path for the reference path *F*_*0*_. This interpolation step was not done for the one Tristan albatross that had a base sampling rate of 2-s. Hermite/cubic splines or Bézier curves might be better options when interpolating seabird paths [66], but at such short (2-s) intervals linear interpolation was acceptable as we do not expect large fluctuations in flight speed at such short intervals. The total distance of *F*_*k*_ was divided by that of *F*_*0*_ to get the proportional distance at different sampling intervals (PD_*k*_), where PD values > 1 indicated an overestimation and < 1 an underestimation of distance. This process is shown in Fig. 2 where sub-sampling of a simulated path has a PD value of 1.12 (i.e. 12% overestimation of distance) at 1-s interval and a PD value of 0.98 (i.e. 2% underestimation of distance) at a 3-s interval.

**Fig. 3:**
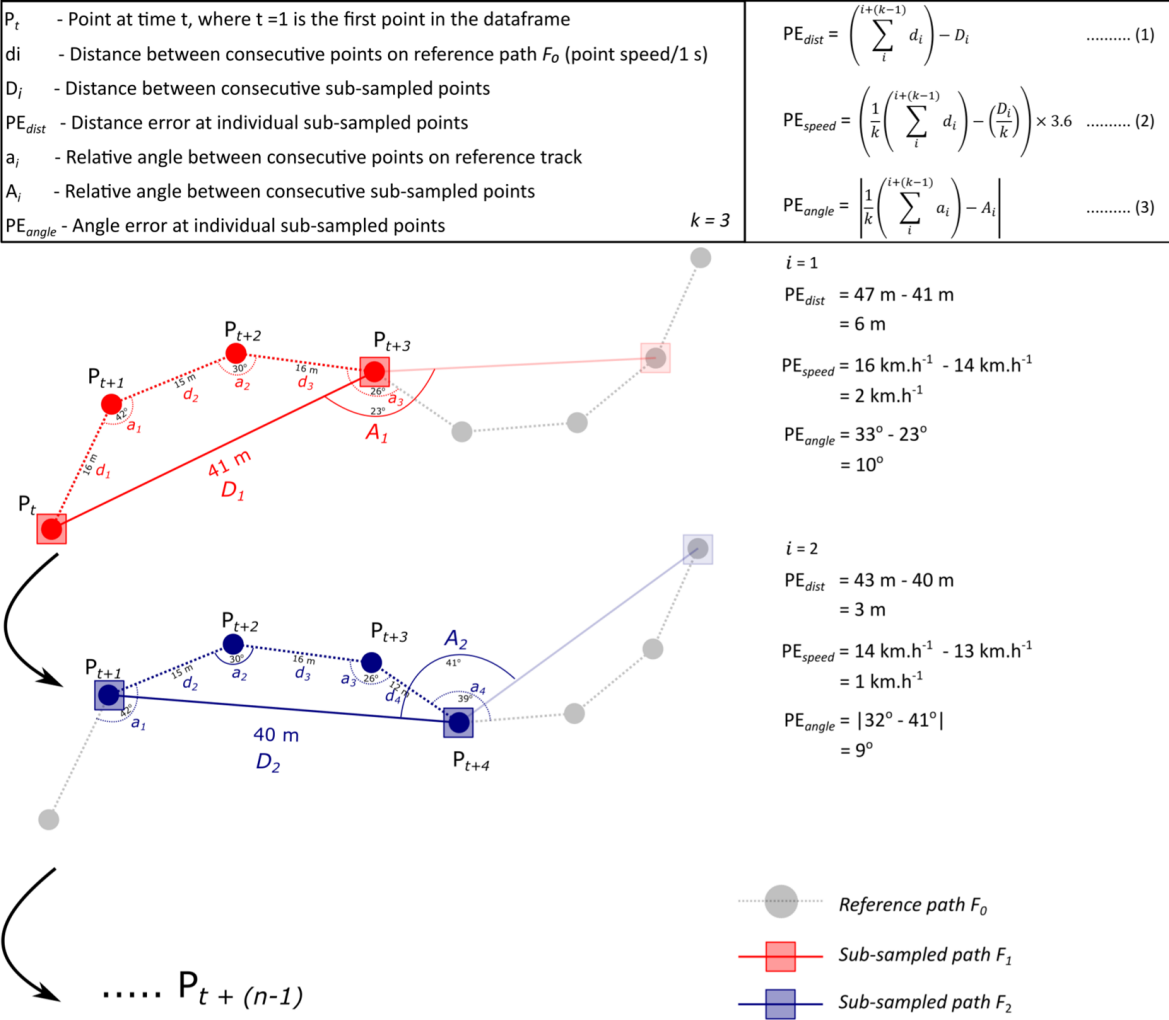
Example of how point error estimates were calculated for two time steps at a sampling interval of *k* = 3 s. These point errors represent the bias associated with metrics commonly extracted from animal tracking data

Point errors (PE) were calculated for each sub-sampled point (Fig. 3) for distance (PE_*dist*_), speed (PE_*speed*_), and relative turning angle (PE_*angle*_). These point errors are not to be confused with measurement errors commonly associated with tracking data (e.g. dilution of precision, DOP values [23]), but rather is a measure of the bias associated with varying sampling rates. This was done as the metrics associated with individual points are important when state-space models are used to estimate behavioural states. Figure 3 shows this process for two consecutive time steps where a simulated path is sub-sampled at a 3-s interval.

PE_*dist*_ is calculated by subtracting the total distance between the sub-sampled points from the total distance of the reference path (*F*_*0*_) falling between the sub-sampled points. Note that the distances of the reference path are derived from the point speeds as described above. PE_*speed*_ was calculated in a similar manner, where the speed values (total distance divided by time) between sub-sampled points were subtracted from the average point speeds of the reference path between the sub-sampled points. Lastly, PE_*angle*_ was the relative angle between sub-sampled points subtracted from the mean relative angles of the reference path between sub-sampled points (absolute values).

#### Behaviour estimation

To examine the effect that the above biases might have on analysis of seabird behaviour from tracking data, a state-space model was used on an example path at varying sampling intervals. Unfortunately, we could only record a full foraging trip with 1-s sampling interval from one individual (wandering albatross) and the rest of the paths did not have enough points to extract behavioural states at coarser (i.e. > 1 min) sampling rates. The path was sub-sampled at 10-s increments, starting at 10 s and ending at 3600 s. Each sub-sampled point was assigned one of three states (rest, forage or commute) as a function of speed (distance over time) and relative turning angle, using the *depmix* function from the *depmixS4* R package [67], with a Gaussian distribution used for both speed and turning angle. The speed estimates are path lengths at the relevant sampling intervals and not the point speed measurements (as these are only useful at ∼ 1 s sampling intervals). The state with low speed and turning angle was referred to as resting, intermediate speed and large turning angles referred to foraging states, and commuting states were represented by high speed and low turning angles. To estimate the difference between sampling rates, the percentage of points belonging to each state was calculated and kernel density estimates were calculated for each state at respective sampling intervals. The kernel density estimates were produced with the *kernelUD* function (R package *adehabitatHR*) and the smoothing factor *h* was chosen using the “*href*” method which is based on the standard deviation of the x and y coordinates [68]. The 50% distribution kernels produced for each sampling interval were compared using the *kerneloverlapHR* function with the “VI” method. We acknowledge that recent advances in analytical tools have presented methods that are not as sensitive to varying scales [48, 69], but biases associated with previously used methods are still necessary for future comparisons or when these scale-sensitive methods are used in current research [49, 70, 71]. Values are reported as means ± SD unless otherwise stated. All analyses were performed within the R software environment [72].

## Results

### Stationary loggers

The ten static GPS loggers produced 380 248 points that were used to determine the error of the units. The loggers performed well, maintaining the 1-s sampling interval for 98% (*n* = 374 265) of the time and the rest (2%, *n* = 5983) at 2-s intervals. The loggers erroneously recorded movement only on a few occasions (0.5%, *n* = 1969 points; Fig. S1). The cumulative distance error (522 ± 618 m) was significantly larger than error from point speeds (156 ± 210 m; *t*_*18*_ = 2.44, *p* = 0.025). Similarly, the mean error per second (0.018 ± 0.021 m) was higher than point speed error (0.006 ± 0.007 m; *t*_*18*_ = 2.144, *p* = 0.046). It has to be noted that our sample size was relatively low and these significance tests have to be taken with caution, but these were in line with previous studies suggesting that point speed is a valid proxy for distance at short intervals [59]. The maximum recorded errors were 51 m and 3 m from distance and point speed estimates, respectively (Fig. S1).

### Tracked birds

Useful data were obtained from 28 individuals from nine different species: 27 birds generated 1 591 743 points at 1-s intervals and one bird 28 201 points at 2-s intervals (Table 1, Supplemental Fig. S2). Overall, the devices performed well, maintaining a 1-s sampling interval 89.7% of the time (1 427 284 points) and 99.6% of points (1 584 986) falling within a 2-s interval. Data gaps > 2 s were rare, and usually occurred at the start and/or end of a path. One individual, that was tracked for a complete foraging trip (2.8 days), had several isolated ∼ 250 s data gaps most likely caused by interference from additional loggers deployed with the GPS. A total of 429 individual flights were isolated with 124 of those lasting more than 1 h, providing 293 h of flight for the sub-sampling analysis. Of the 124 flights, 64 (143 flight hours) were classified as ‘straight’ and 60 (150 flight hours) as sinuous flight.

### Sub-sampling

**Fig. 4:**
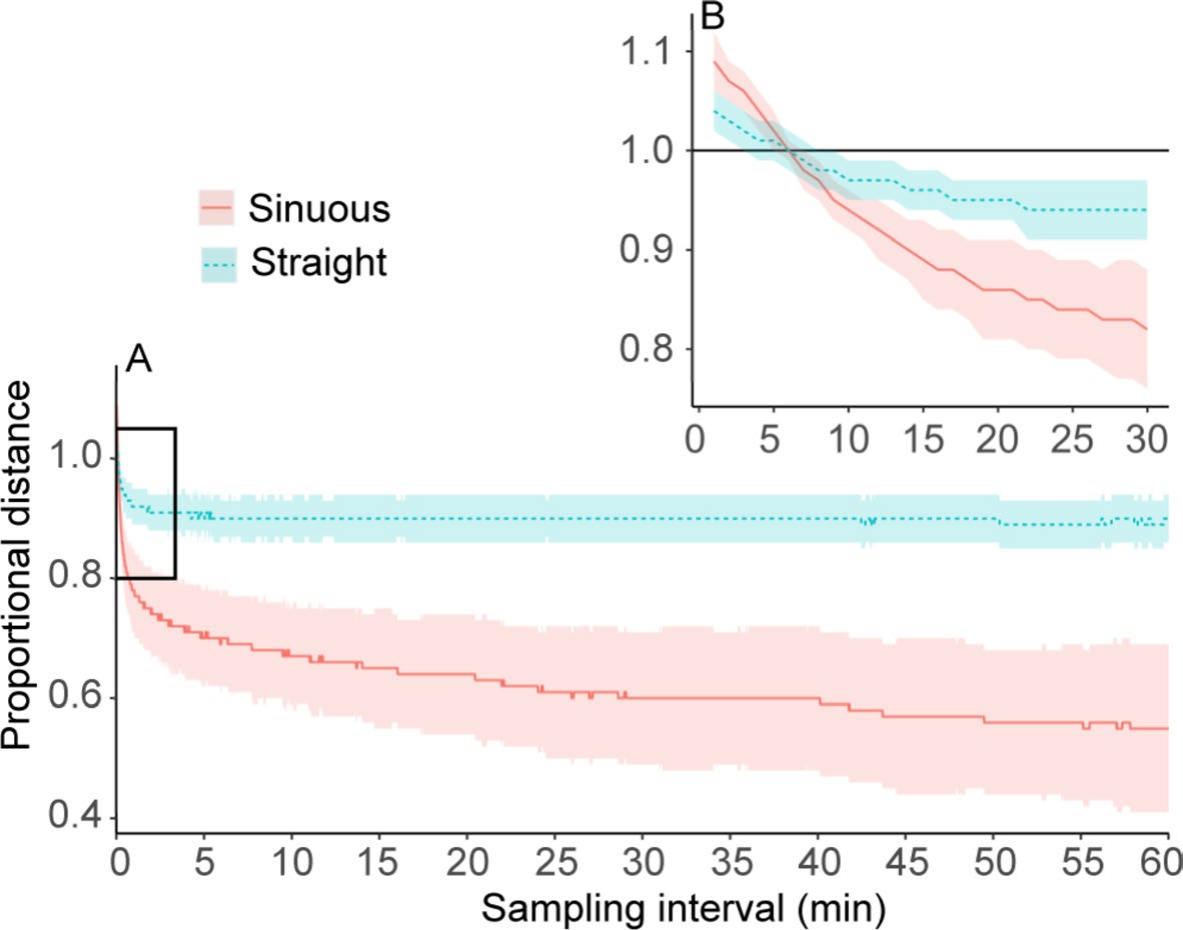
(**a**) Proportional distance estimates for straight and sinuous flights at increasing sampling intervals. (**b**) Zoomed in portion showing the same curves as in a), but only up to 30-s interval

**Fig. 5:**
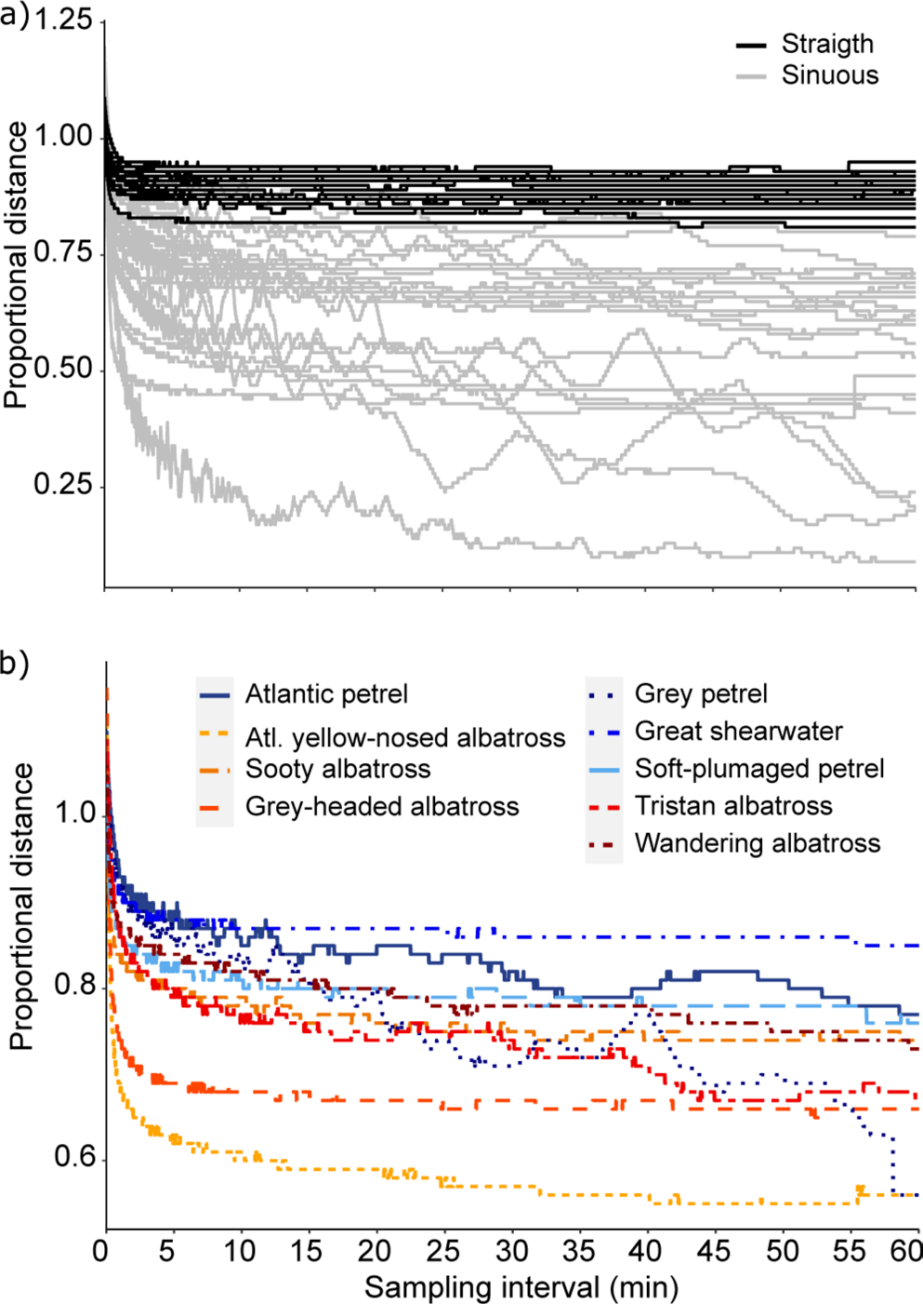
Proportional distance estimates of flying seabirds tracked at 1-s sampling interval. Curves are means for (**a**) each individual separated into straight and sinuous flights and (**b**) averages for each species

On average, the total distance of sub-sampled flights was overestimated (PD > 1) up to *k* = 6 s for both sinuous (9% overestimate) and straight (4%) flight types, after which it was underestimated (PD < 1; Fig. 4). From *k* = 6 s to 30 s, the degree of underestimation increased rapidly, after which it levelled out around *k* = 60 s for both flight types, but the bias for sinuous flights continued to increase steadily as *k* increased up to 3600 s (Fig. 4). The average underestimate for k > 60 s for straight flights (10%; PD = 0.90 ± 0.04) was much less than that of sinuous flights (38%; PD = 0.62 ± 0.05). Individual variability in PD also was much greater for sinuous flights than for straight flights (Fig. 5a) and although sample sizes were small, it is possible that species-specific flight patterns may also influence biases (Fig. 5b and Supplementary Fig. S3). The flight of Atlantic yellow-nosed albatrosses was significantly more sinuous than any of the other species (Fig. 5b and Supplementary Fig. S3; *t*_*19*_ = 2.7, *p* = 0.014). This might simply reflect a high proportion of sinuous foraging paths, but two of the four yellow-nosed albatross paths seemed to be commuting flights (Supplementary Fig. S4) and the low sinuosity is possibly as a result of erratic fine-scale movement during cruising flight.

**Fig. 6:**
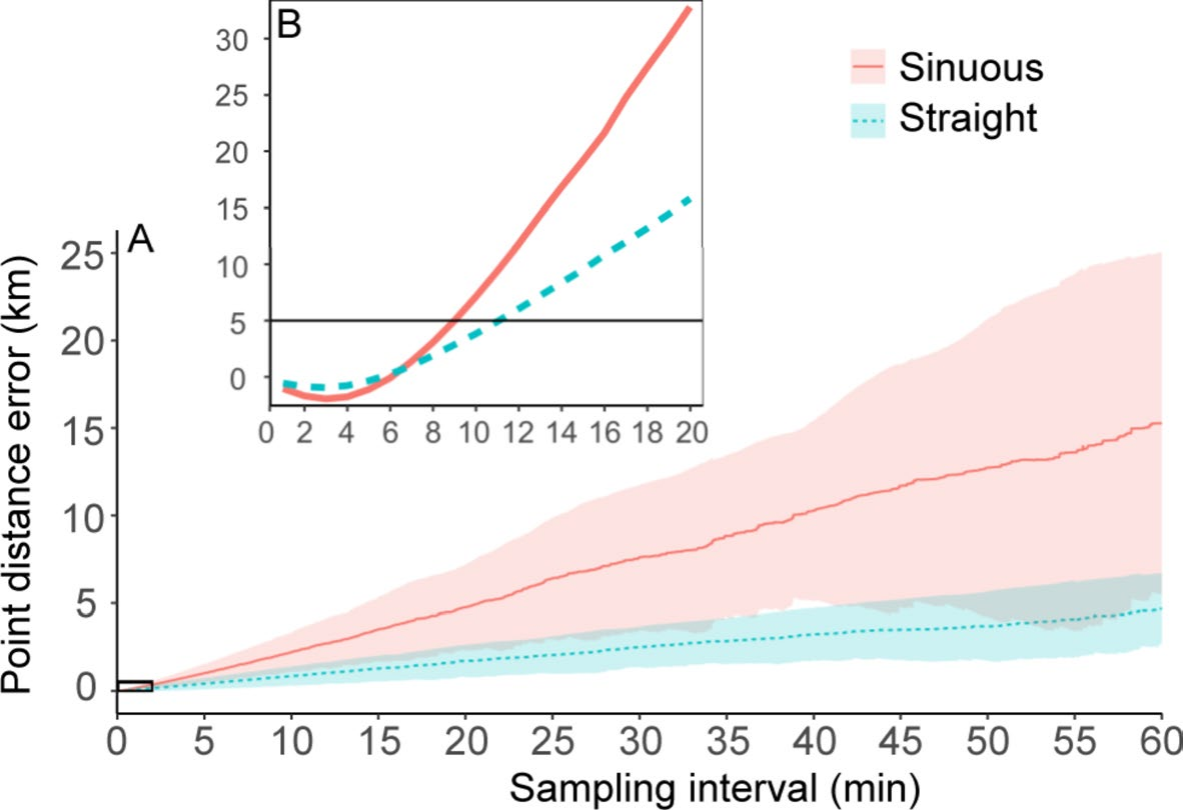
(**a**) Mean ± SD point distance error (*PE*_*dist*_) estimates for sinuous and straight flights of all tracked birds. (**b**) Zoomed in section of a) with means only and a solid line shown at an error of 5 m, within the positional error margins of the GPS loggers used

**Fig. 7:**
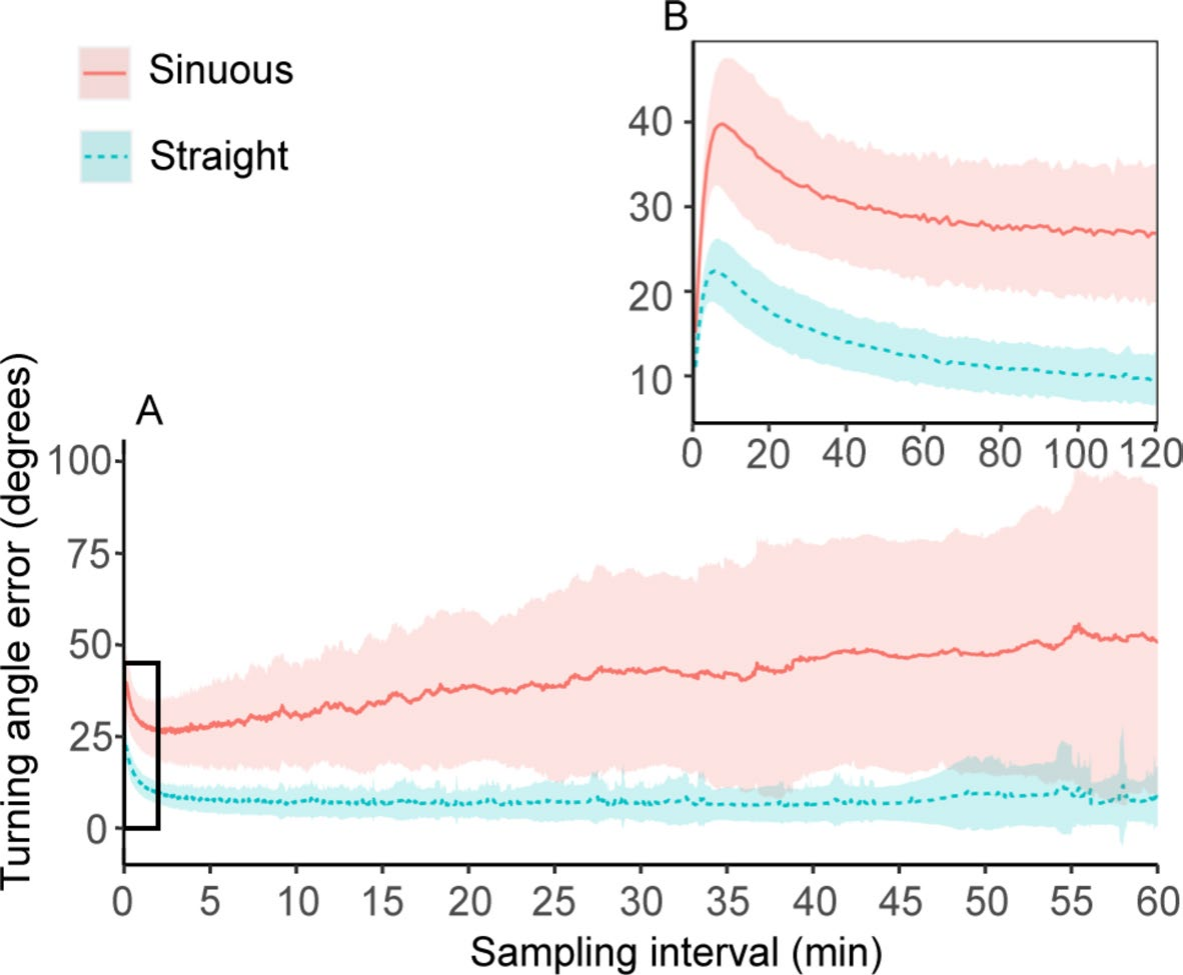
(**a**) Mean ± SD point error for turning angles (*PE*_*angle*_) of flights at varying sampling intervals. (**b**) Zoomed in section of a)

The average distance error for individual points (PE_*dist*_ ) was < 5 m up to *k* = 9 s for sinuous flights and *k* = 11 s for straight flights, with a steeper slope for sinuous flights ending at a value of 15.2 ± 9.8 km for *F*_*3600*_ compared to a value of 4.7 ± 2.0 km for straight flights (Fig. 6). The average speed error for individual points (PE_*speed*_) curves were similar to the PD curves in Fig. 4 (seeing that the speed was derived from distance), with a maximum PE_*speed*_ of 12 ± 5 km·h^− 1^ for sinuous flights and 8 ± 2 km·h^− 1^ for straight flights at *F*_*3600*_ (Fig. S5). The average relative angle error for individual points (PE_*angle*_) increased sharply up to *k* = 10 s (where it reached a maximum value for straight flight; 22 ± 4°), after which it decreased up to *k* = 120 staying steady for straight flight but increasing steadily for sinuous flights with a maximum value of 57 ± 38° at *F*_*3600*_ (Fig. 7).

### Behavioural estimation

**Fig. 8:**
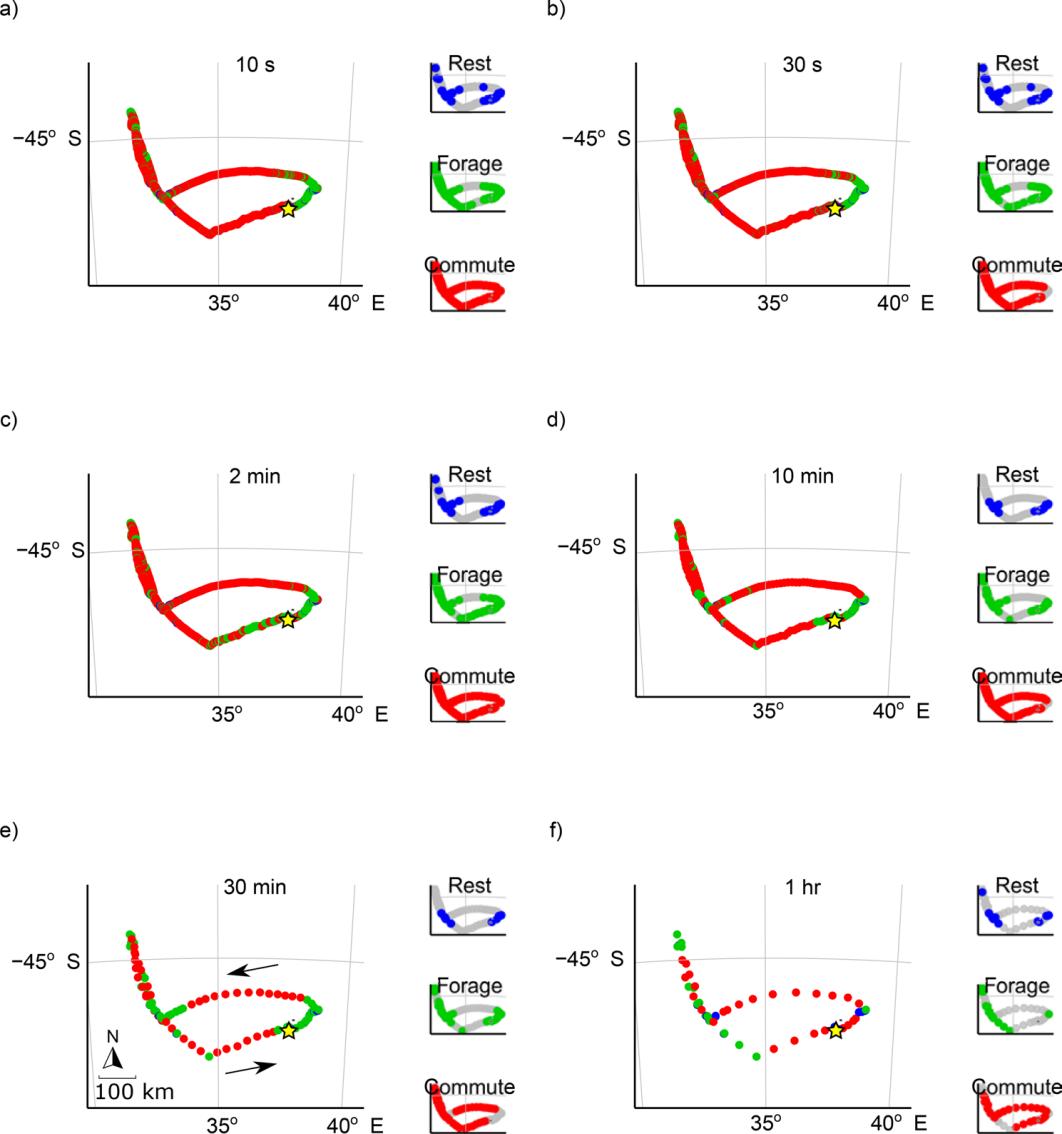
Example of the same state-space model run on a 37-hr foraging trip of a wandering albatross at different sampling intervals (10-s, 10-min, 30-min, and 1-hr), estimating commuting, foraging, and resting states. The star indicates the location of Marion Island

**Fig. 9:**
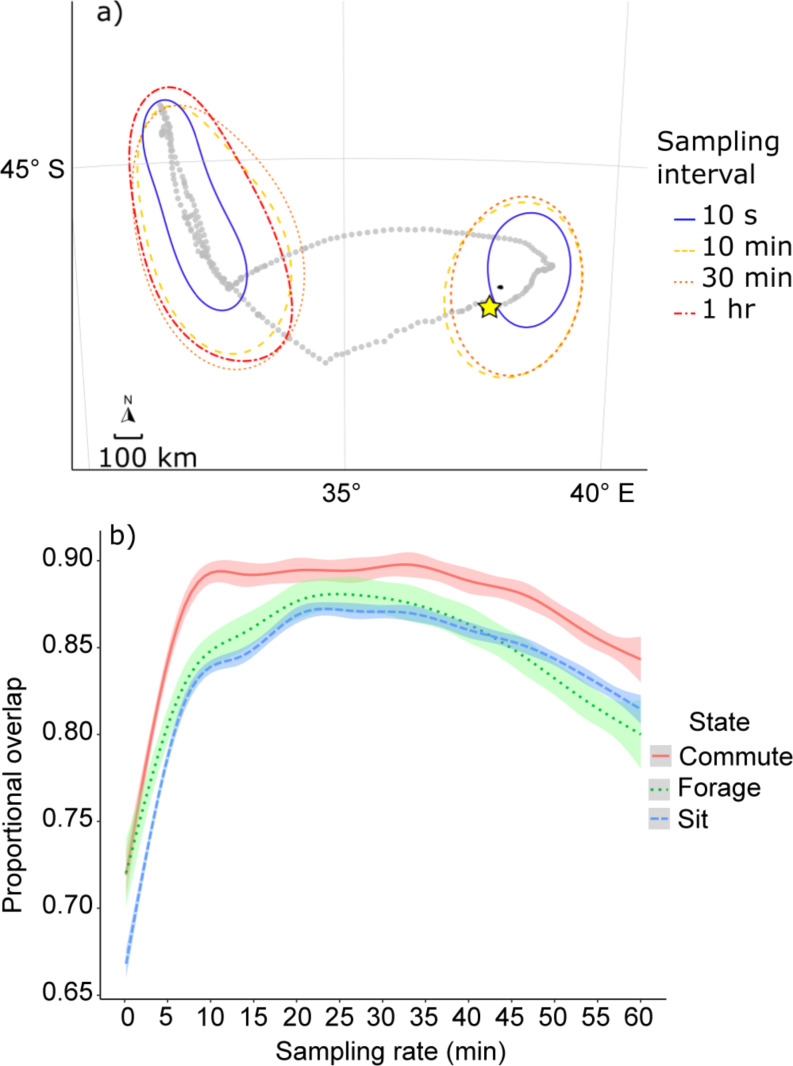
Effect of sampling rate on identification of behaviour states using a state-space model on the same data as Fig. 8. (**a**) Shows kernel density estimates of points identified as “foraging” at four different sampling rates. (**b**) Shows the proportional overlap of kernel density estimates of respective behavioural states at varying sampling intervals

State-space modelling, where speed and turning angle were used to determine states, had variable results depending on the value of *k* (Fig. 8, Fig. S6). At ‘high’ sampling rates (< 300 s) the states were most likely overrepresented by commuting and foraging states, with little distinction between the two (Fig. 8a). At moderate sampling rates (10–30 min intervals) the states became more distinct and areas associated with each behaviour could be clearly seen (Fig. 8b-c). At the lowest sampling rate (1 h), the states were still distinct, but seemingly different from those identified from moderate sampling rates (Fig. 8d). The same trends were found from the kernel density estimation of foraging states at varying sampling intervals where the states identified at moderate sampling rates were most stable, while fine (< 5 min) and coarse sampling rates (1 h) had lower overlap with the rest of the points (Fig. 9). These stable periods are not necessarily more accurate reflections of behavioural states, but rather show blurring of behaviours in space as sampling intervals become coarser, thus changing from specific foraging sites to general foraging areas. The rapid change up to 5 min (Fig. 9b) suggests the derived states may change perspective dramatically at these finer sampling intervals. The reason for these variations becomes clear from Fig. S7, where the variation in speed and relative angle at different *k* values are shown. At lower sampling rates speed has a bimodal distribution, but this distribution flattens out as sampling interval increases, making it more difficult to distinguish different states.

## Discussion

The results show the effect that sampling interval has on information obtained from GPS tracking data in flying seabird behavioural studies. Although the sample sizes were small, data from a wide range of species (varying in size and breeding location) were obtained and analysed to highlight the various factors that should be considered when interpreting GPS tracking data.

### Device performance

As noted by [24], the low-cost GPS loggers used in this and many other seabird tracking studies had similar performance to more conventional commercial devices at all sampling intervals. The manufacturer specifications for the SiRFstarIII chipset claim it can obtain GPS fixes at a maximum rate of 1 Hz with 2.5 m horizontal position accuracy. However, in practice, the accuracy and fix acquisition rate of GPS loggers can be affected by many factors. Several studies have detailed the efficacy of low-cost GPS loggers in varying environmental conditions and generally the horizontal positional accuracy is closer to 5 m, depending on obstruction above the GPS (e.g. tree cover) and position of the antenna [23, 24, 73]. The fix success rate of low-cost GPS loggers is often close to 100% [23], which was the case for the stationary loggers tested in our study, but loggers deployed on seabirds had a lower fix success rate (90%). Weather conditions in the sub-Antarctic are harsh and could affect the fix rate, and some loggers were deployed with additional loggers (cameras, IMUs), which might have interfered with their ability to obtain fixes. Most GPS loggers record information regarding fix quality [23], but downloading these data is not the default setting and it is often not downloaded (as was the case in our study). Additionally, at very high sampling rates (∼ 1 Hz), paths estimated from low cost GPS loggers are subject to measurement error [59], where error associated with their accuracy (usually ± 5 m) compounds, resulting in inflated distances between points. However, point speed values recorded by the GPS loggers is derived from Doppler shift and are not implicated in the measurement error [60]. Our results confirm that the biases associated with point speeds are much smaller than biases from displacement measurements for stationary loggers. Point speeds can thus be used as a proxy for distance at 1-s sampling rates to create reference paths with smaller biases.

### Fine-scale use

When estimating travel distances of flying seabirds from successive GPS locations, sampling intervals > 10 s will likely result in underestimation of the distances. For example, a great shearwater tracked with a 1-hr sampling interval on a three week foraging trip may report a total distance of 11 000 km [13], but the actual distance may be closer to 12,100 km (10% underestimate) or even up to 15 400 km when the flight is extremely sinuous (40% underestimate). In reality, longer foraging trips may have large portions of commuting where flight may be straighter [42] and at hourly intervals total distance travelled will most likely be underestimated by somewhere between 10 and 40%. Our results are limited to procellariiform seabirds, mostly using a curved flight path associated with dynamic soaring flight. Seabird species that rely more on flapping flight have less sinuous flight paths, and fine-scale location sampling may be largely irrelevant when tracking their movement.

When estimating path length from successive GPS locations, sampling intervals > 60 s will likely result in path length biases of up to 5 km (at 1 h intervals). Sinuous (often foraging) flights could have a much larger bias, up to 15 km for path lengths at hourly intervals. When sampling at high frequencies (< 60 s intervals) the bias becomes smaller, but only at rates < 10 s does it reach biases that are within the accuracy range of the loggers (i.e. path length error < 5 m). However, when sampling at rates < 6 s, the path lengths and total distance will likely be overestimated due to measurement error by up to 4% and 9% for straight and sinuous flights, respectively. To avoid overestimation at these very frequent sampling intervals, point speeds should be used as a proxy for distance.

Points speed and heading recorded by GPS loggers are measured using Doppler shifts and previous studies have shown speed to be within 0.01 m·s^− 1^ of actual speed on straight paths and within 0.02 m·s^− 1^ on curved paths [74], while heading is accurate to 0.01° according to manufacture specifications (but likely less accurate in practice). However, these are only useful at very high sampling rates and because they are instantaneous measurements, could provide values that are not representative of the overall movement (especially for heading). This will likely be the case for dynamic soaring seabirds such as most albatrosses and petrels that constantly change speed and heading during flight [75–77]. However, Safi et al. [64] suggested that point speed and heading estimates might be more informative than using successive locations, even at coarse sampling rates. These authors also suggested using short bursts of 1-s sampling rates where point speed measurements could not be recorded by loggers. Our results suggest that this might be a plausible solution as measurement error only becomes a problem when compounded over many hours. In short bursts, the measurement error of high frequency (∼ 1 Hz) data should be small enough to be comparable with point speed measurements (see Fig. 6). Other options include varying sampling rates according to external triggers or power availability [53, 78]. However, this has to be done with caution because our results suggest that estimating speed from successive locations at high sampling intervals could overestimate speed and result in large biases in turning angle. Measurement error could result in erroneous conclusions from tracking studies and it might be beneficial to exclude points that are very close to each other in time [22, 79]. Where fine-scale paths are needed, specialized loggers that incorporate satellites from several systems and record raw signal data are better to obtain the required accuracy [80, 81]. Alternatively, dead-reckoning complemented with intermittent GPS locations could produce paths with high accuracy [54], as has been done with humpback whales *Megaptera novaeangliae* [82]. However, dead-reckoning for dynamic soaring seabirds will be challenging as they constantly change their heading and may be subject to wind drift [36, 83] that will not be easy to estimate without matching high resolution wind data.

### Inferring behaviour

Although sample sizes were small, there seemed to be differences in biases among species. Most notably, Atlantic yellow-nosed albatrosses had extremely tortuous fine-scale flight patterns, with their flights significantly more sinuous than other species. Two yellow-nosed albatross paths were not obviously related to foraging as the overall paths were relatively straight, but the fine-scale movement was tortuous. In contrast, great shearwaters performed very straight commuting flights with smaller biases in total distance travelled. These shearwaters perform long foraging trips during incubation and alternate long and short trips during chick-rearing periods [13, 84]. These extraordinary chick-rearing trips often have high travelling speeds while commuting between foraging areas where the birds travel up to ∼ 14 000 km [13]. However the total distance reported by Schoombie et al. [13] is most likely an underestimate as they used hourly GPS sampling rates and the actual distance is probably closer to at least 15 000–17 000 km (10–20% underestimate) when accounting for sampling rates. Location data are now frequently used as management tools, for example, identifying areas of concern in the Southern Ocean [2, 6]. Such studies could underestimate the areas that are being used by the animals that they tracked. Likewise, energy expenditure derived from location based movement data will likely be inaccurate if scale-sensitive metrics are not accounted for [33].

Body size did not have a marked effect on biases as the largest (wandering albatross) and smallest species (soft-plumaged petrel *Pterodroma mollis*) had similar proportional distance curves (Fig. 5b). However, wandering albatrosses had the largest sample size (*n* = 8) while only two soft-plumaged petrels were sampled. The behaviour of the birds (i.e. foraging vs. commuting) undoubtedly contributes to observed differences (or lack thereof) between species. Nonetheless, studies comparing several species of seabirds using coarse GPS sampling intervals might be at risk of comparing behaviours that are not directly comparable as paths from different species may be affected differently by sampling interval. Determining the effects of sampling intervals by having a subset of loggers with higher sampling rates might be beneficial for individual studies, as shown by Tarroux et al. [36].

When using location data to infer behavioural states, bias in relative turning angles can completely change the inferred behaviours [51, 79]. Biases associated with turning angle are very sensitive to sampling interval of GPS paths. Particularly interesting is the steep increase in error from *k =* 1–10 s with a local maximum at *k* = 10 s for both sinuous and straight flights. Turning rates are often used in flying seabird studies to differentiate foraging, commuting and resting periods (e.g. [30, 32, 45]). The large variation of inferred behaviour at high sampling frequencies (< 300 s intervals) is most likely as a result of larger bias in turning rate at these frequencies. The inference of behaviour might again become unreliable at low sampling rates > 30 min intervals, as the bias associated with path length increases. High levels of uncertainty in predictor variables (i.e. speed and turning angles) may significantly impact models using such predictors to infer behavioural states of seabirds and different conclusions can be drawn from the same path at varying sampling intervals [51]. Location only data could be useful to infer behavioural changes or identify foraging areas at broad scales [30] but inferring fine-scale behaviour using coarse location data risks resulting in erroneous conclusions.

The impact of measurement error on SSMs have received much attention in recent years, and in particular, [33] have warned against the use of SSMs without a thorough understanding of the statistical limitations. We used a Gaussian distribution for both speed and turning angle when running the SSMs, where a Gamma (speed) and von Mises (turning angle) distribution may have been more appropriate [85]. Implementing models with these distributions is challenging as they require adequate starting values when initiating the model [85, 86], but this may be necessary to obtain accurate behavioural states from tracking data. At high sampling rates, zero-inflation became a problem when path lengths (speed) could be zero (see Supplemental Figure S6), but more recent applications of SSMs allow for zero-inflation [e.g. 86]. Likewise, when calculating kernel density estimates, high sampling rates could lead to auto-correlation induced biases, and methods that allow for autocorrelation are recommended [e.g. 87]. Behaviour coupled with distribution can provide informed distribution maps [88], but behaviour derived from location estimates are probably not informative enough and a multi-sensor approach might be more beneficial [89]. For example, fine-scale GPS locations (5-s interval) in conjunction with cameras showed fine-scale behaviour of Cape Gannets *Morus capensis* [90]. However, when GPS loggers are coupled with ancillary loggers lower sampling rates may be acceptable when fine-scale movements are adequately described by the accompanying loggers, e.g. [91].

In some cases the need for fine-scale data can be overemphasized [25]. When fine-scale data are not necessary to answer a certain research question, it might better to use coarse sampling rates (i.e. hourly fixes) and reduce logger mass by using smaller batteries, consequently reducing the load the study species has to carry. For example, coarse sampling rates can give information regarding “hotspot” areas that could be associated with foraging, but these areas are easily identified by using simple kernel density estimates, negating the need for complex modelling [30]. Recent description of analytical tools that are less sensitive to varying sampling rates have been described [48], but these are limited by the models they can fit and could be computationally expensive to perform [92]. Where fine-scale data are required, using loggers with higher accuracy, or a multi-sensor approach might be better. Nonetheless, we urge researchers to carefully consider sampling rates when analyzing tracking data and to use appropriate methods to take the relevant biases into account. Our study is restricted to flying seabirds, but the results may be applicable to other seabirds or even terrestrial animals where GPS path lengths are used to derive metrics and infer behaviour.

### Electronic supplementary material

Below is the link to the electronic supplementary material.

Supplementary Material 1

## Data Availability

The datasets used for analysis during the current study can be accessed on the University of Cape Town’s ZivaHub repository at: https://doi.org/10.25375/uct.26830189.
