# Supplementary material for: The efficiency of detecting seabird behaviour from movement patterns: the effect of sampling frequency on inferring movement metrics in Procellariiformes
Source: Mov Ecol. 2024 Sep 2;12:59. doi: 10.1186/s40462-024-00499-1 (PMC11370088; doi:10.1186/s40462-024-00499-1)
Supplement: Supplementary file 1 — Supplementary Material 1 [file 40462_2024_499_MOESM1_ESM.docx]

# The efficiency of detecting seabird behaviour from movement patterns: the effect of sampling frequency on inferring movement metrics in Procellariiformes

Stefan Schoombie^A,B,E^, Rory P. Wilson^C^, Yan Ropert-Coudert^D^, Ben J. Dilley^A^, Peter G Ryan^A^

^A^ FitzPatrick Institute of African Ornithology, DST-NRF Centre of Excellence, University of Cape Town, Rondebosch, 7701, South Africa

^B^ Centre for Statistics in Ecology, Environment and Conservation (SEEC), Department of Statistical Sciences, University of Cape Town, 7701, South Africa.

^C^ Department of Biosciences, Swansea University, Swansea SA1 8PP, UK

^D^Centre d’Etudes Biologiques de Chizé, Station d’Écologie de Chizé-La Rochelle Université, CNRS UMR7372, Villiers-en-Bois, France

^E^ Corresponding author. Postal address: Centre for Statistics in Ecology, Environment and Conservation (SEEC), Department of Statistical Sciences, University of Cape Town, 7701, South Africa. E-mail: [schoombie@gmail.com](mailto:schoombie@gmail.com)

## Supplementary material


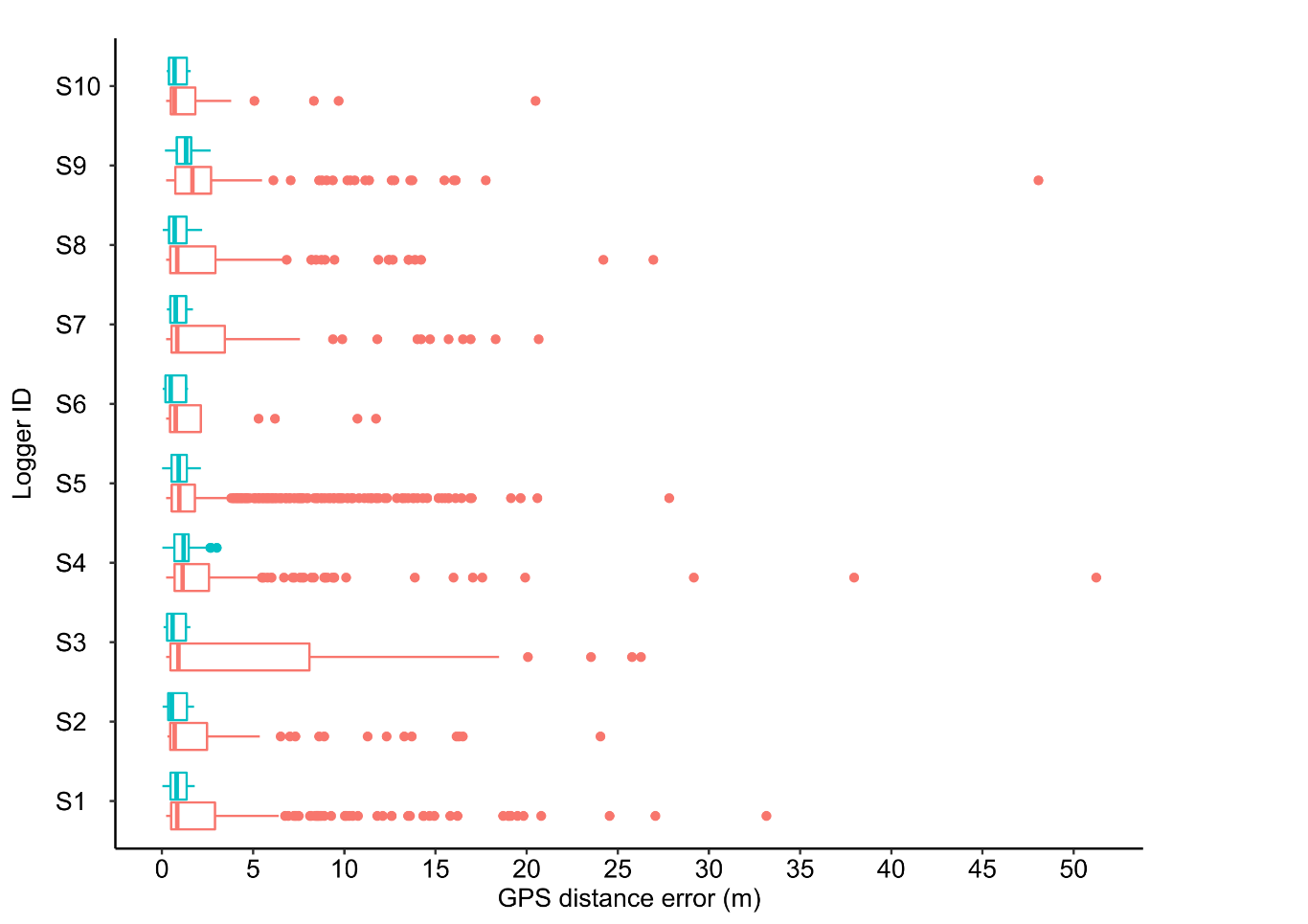


**Fig. S1:** Boxplots of error associated with stationary low-cost GPS loggers recording position and point speed at 1-s intervals for ~12 hours on Marion Island. Values are from points where the distance between points was > 0 m (*n* = 1969 points), indicating erroneous location estimates. Boxplots are shown for error derived from distance (red) and point speed (blue).


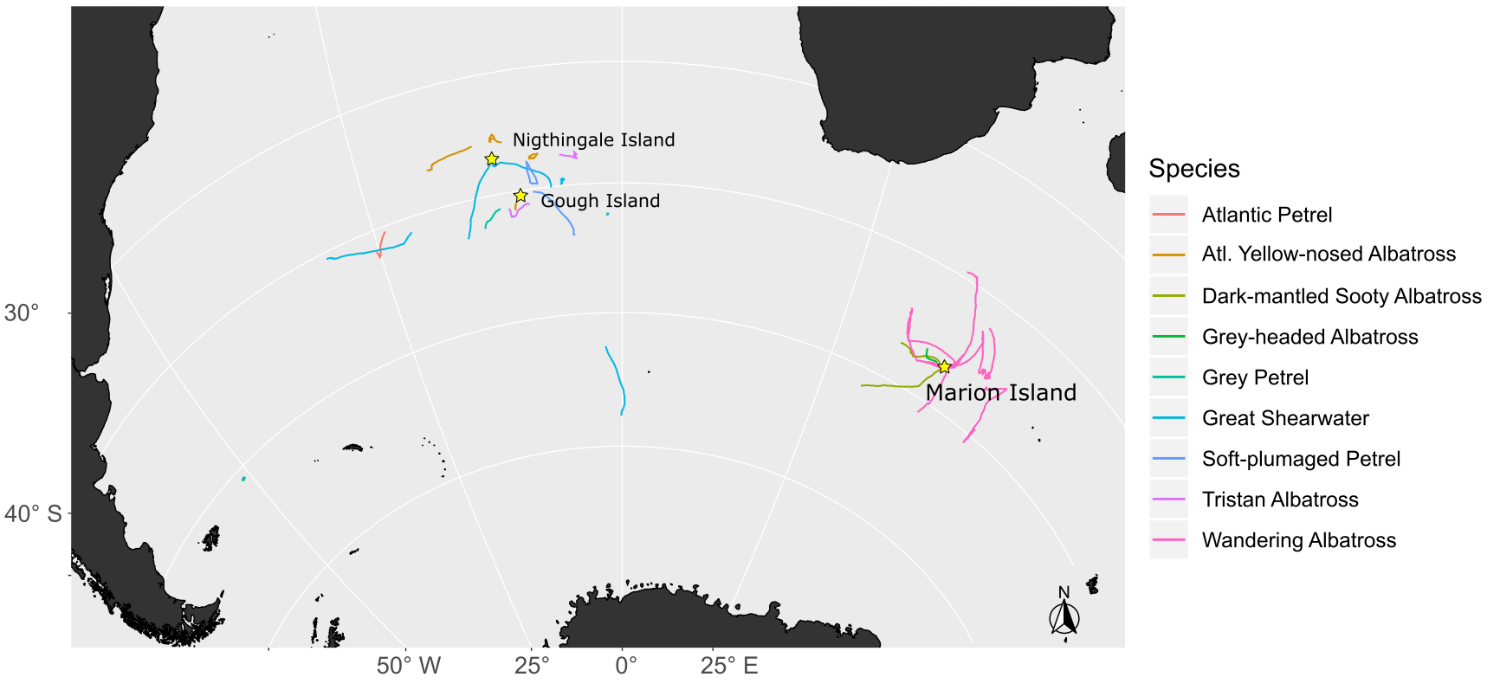


**Fig. S2:** All GPS paths for Procellariiformes tracked at 1-s sampling intervals (2-s sampling interval for one Tristan Albatross) from Marion, Gough and Nightingale Islands.

°


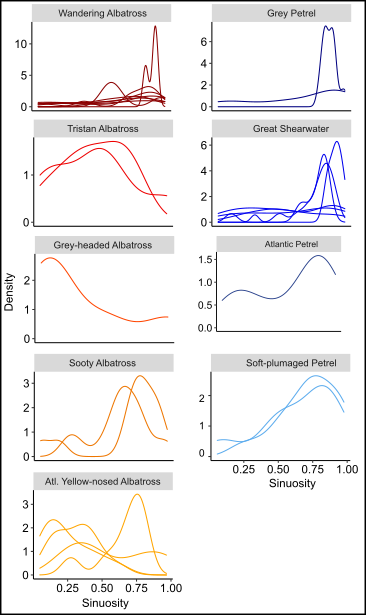


**Fig. S3**: Frequency distribution of flight sinuosity for individuals from respective species tracked with GPS loggers at 1-s sampling intervals (or 2-s sampling interval for one Tristan Albatross).


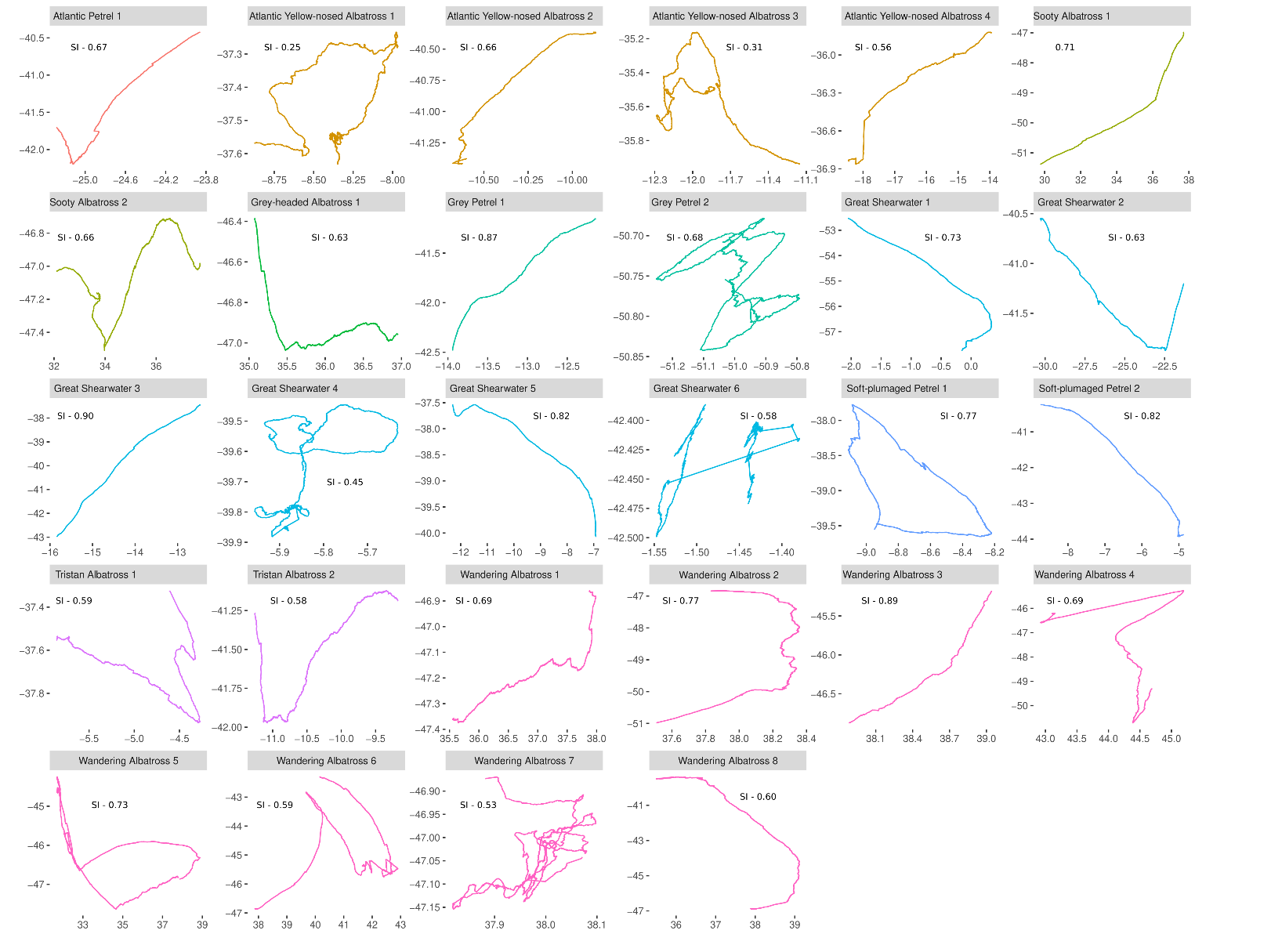


**Fig. S4**: Tracking data from individual albatrosses and petrels tracked with GPS loggers at 1-s sampling intervals (or 2-s sampling interval for Tristan Albatross 1). The raw paths are shown for individual birds with the mean sinuosity index of flights (SI) shown for each track.


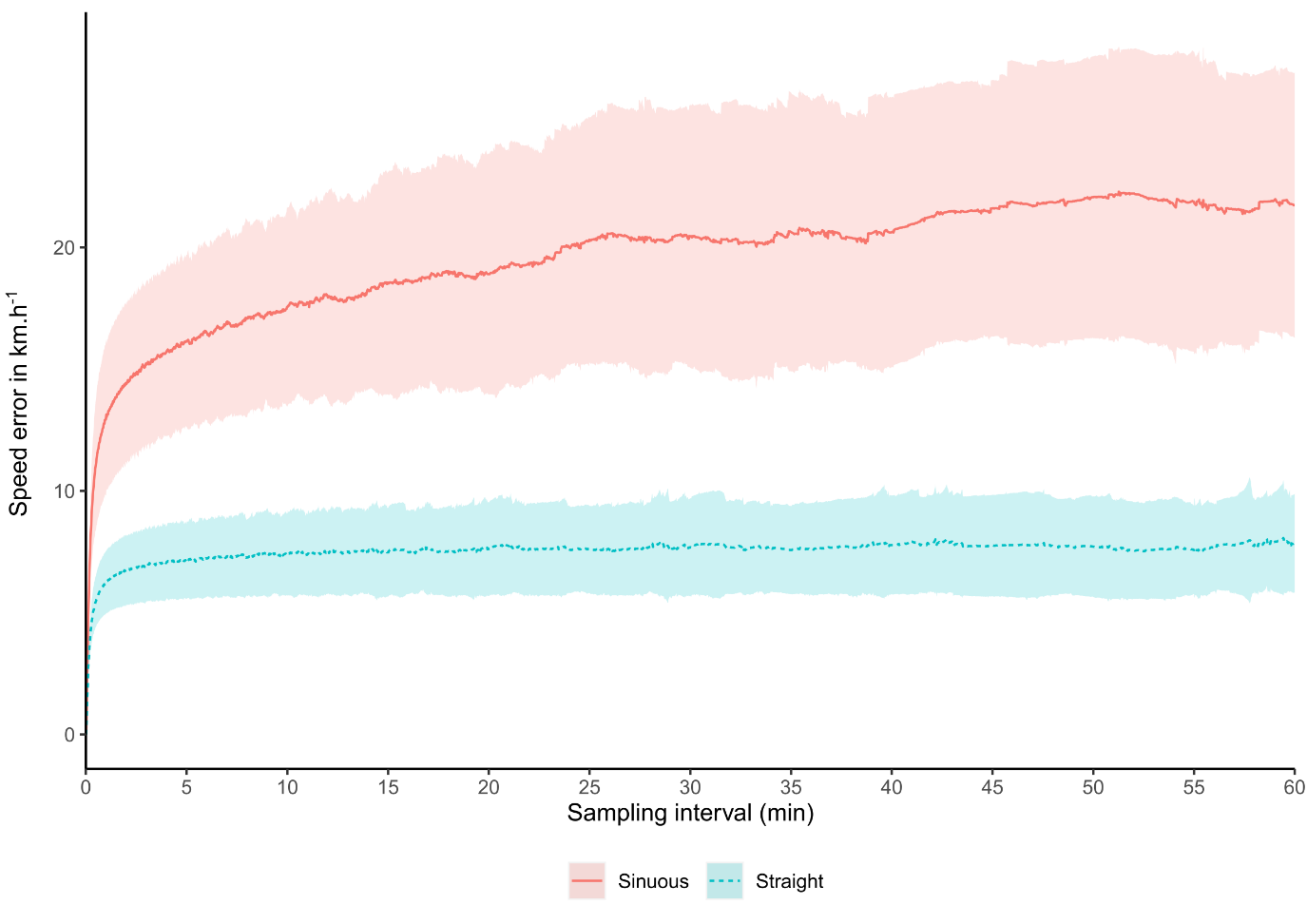


**Fig. S5:** Mean ± SD point speed error (*PE_speed_*) for flights at varying sampling intervals.


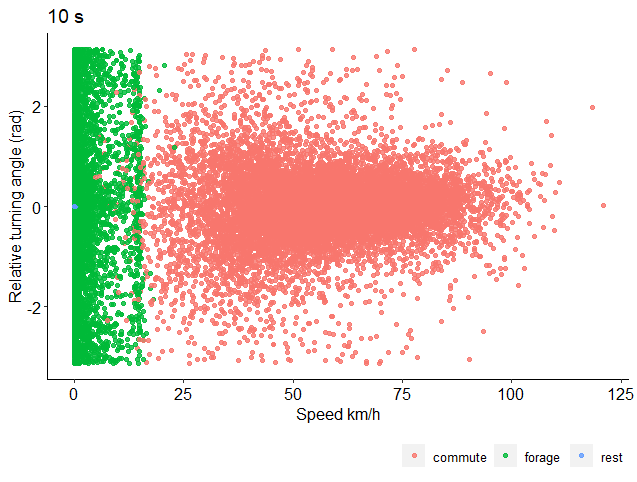

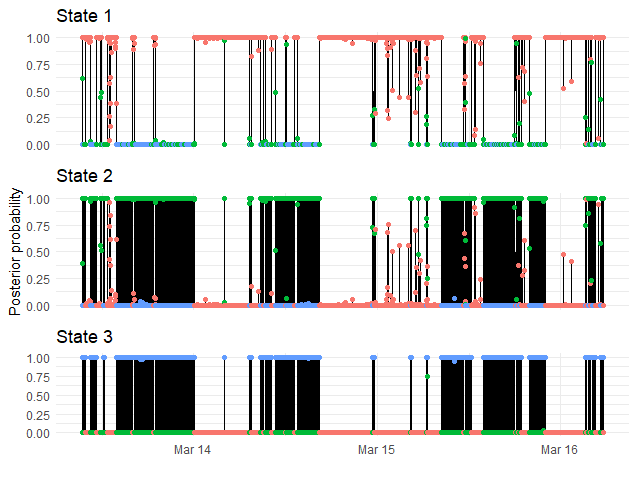

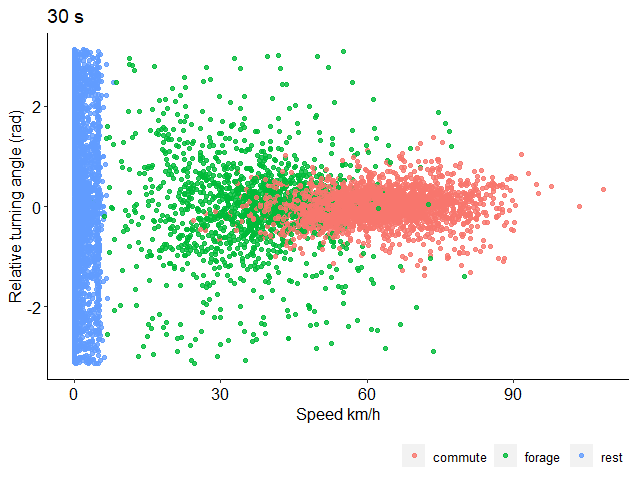

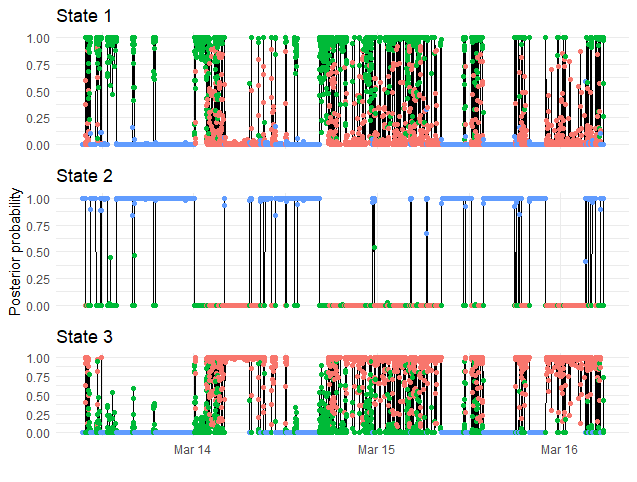

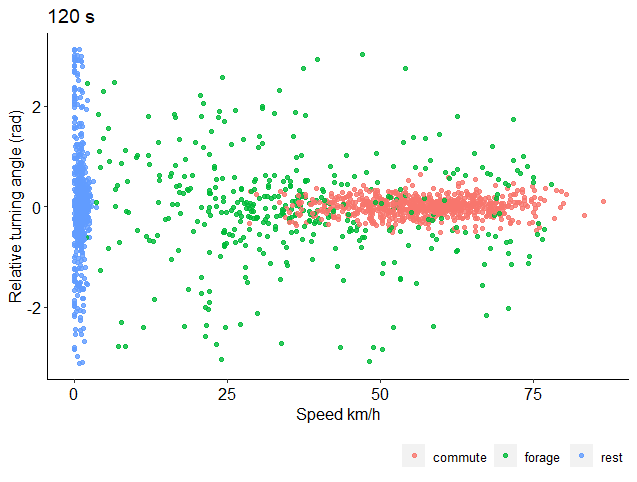

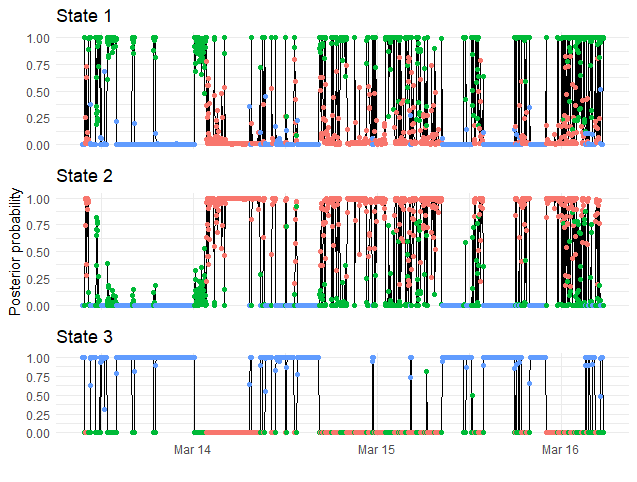

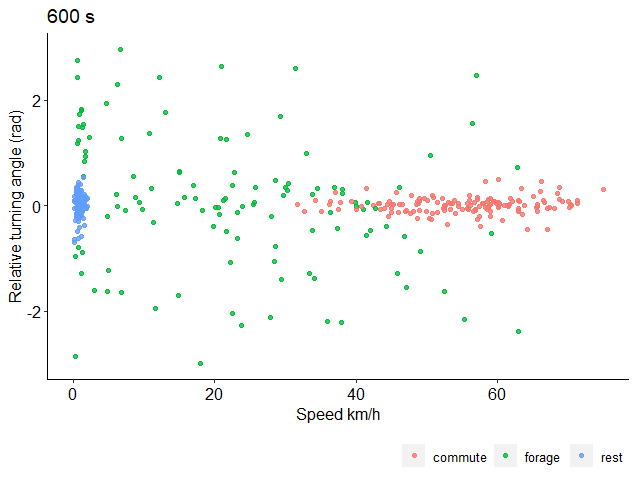

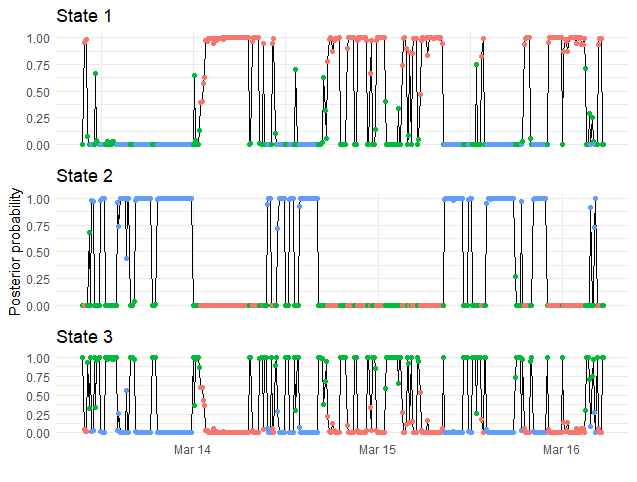

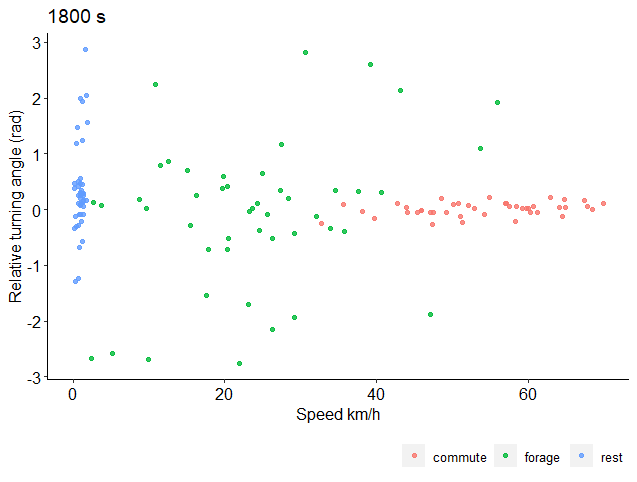

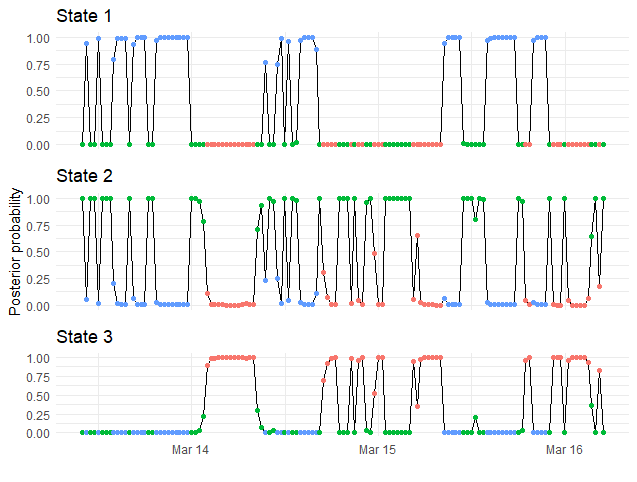

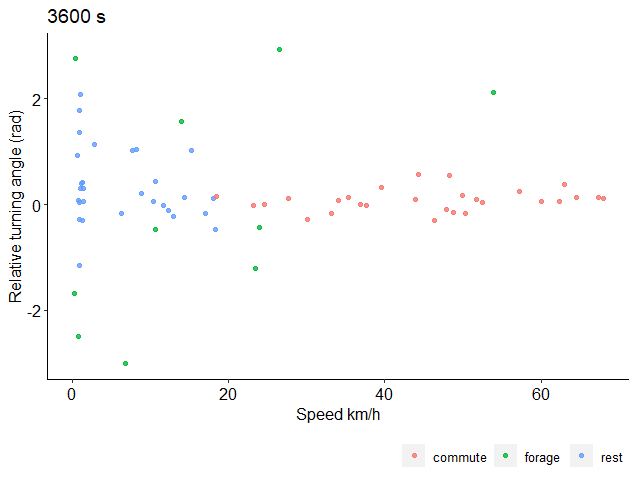

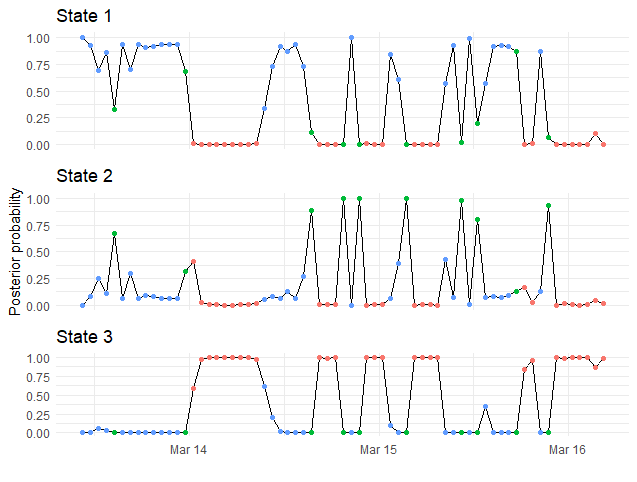


**Fig. S6 (continued form previous page):** Distribution of speed and relative turning angles at varying states together with distribution of posterior probabilities for each state at a range of sampling intervals.


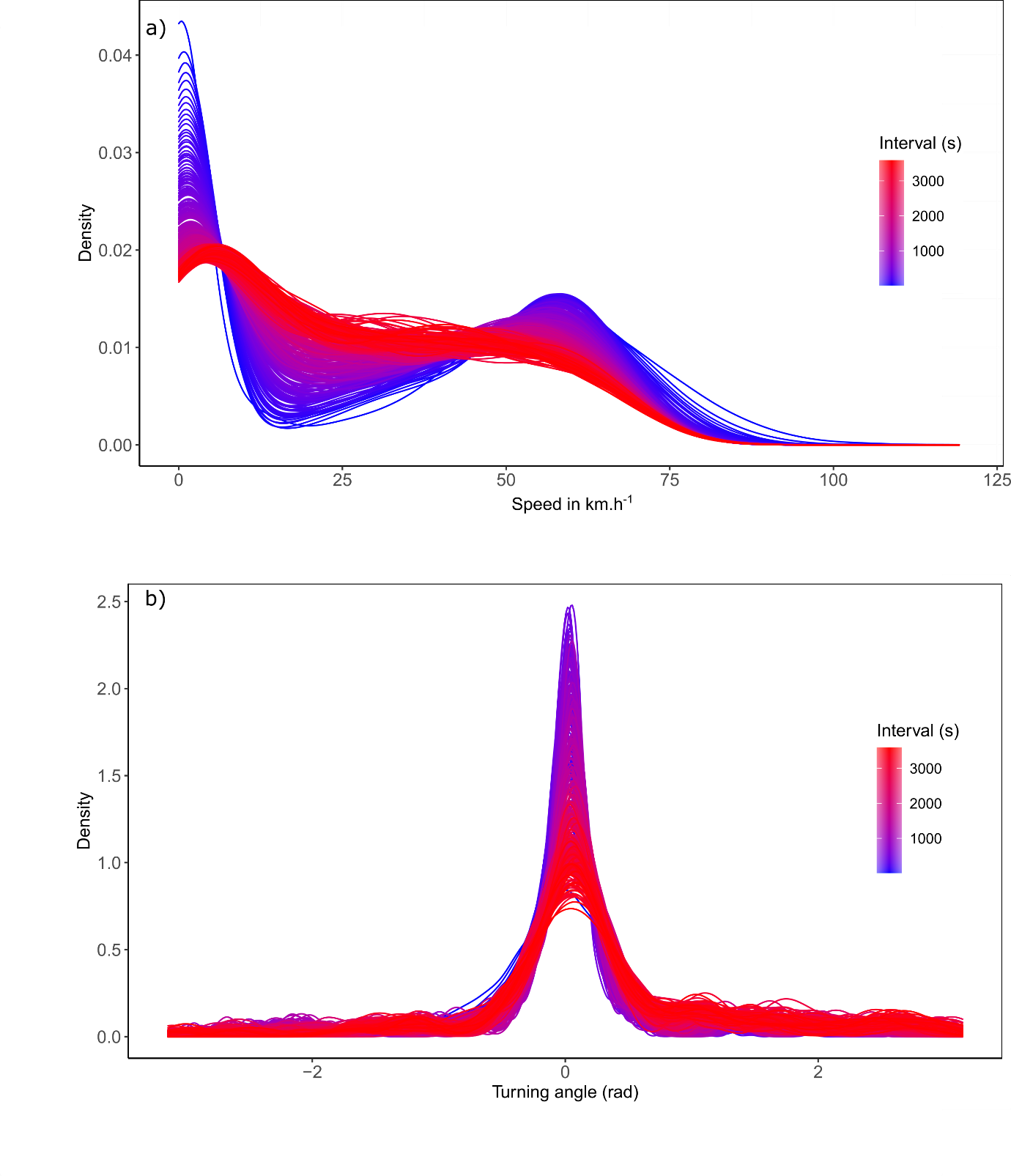


**Fig. S7:** Density plots of **a)** speed and **b)** turning angle at varying sampling intervals for a complete foraging trip performed by a Wandering Albatross tracked with a 1-s sampling interval.
